# The Role of the Extracellular Volume of the Infarction Zone and the Remote Myocardium in Predicting Systolic Dysfunction After the First Myocardial Infarction

**DOI:** 10.3390/diagnostics16162663

**Published:** 2026-08-20

**Authors:** Valentin Oleynikov, Lyudmila Salyamova, Alexander Vdovkin, Natalia Donetskaya, Irina Avdeeva, Inna Babkina, Elena Averyanova

**Affiliations:** 1 Therapy Department, Penza State University, 40, Krasnaya St., 440026 Penza, Russia; l.salyamova@yandex.ru (L.S.); innalevina@mail.ru (I.B.);; 2 Regional Clinical Hospital n/a N.N. Burdenko, 28, Lermontova St., 440026 Penza, Russia; wdovkin@yandex.ru (A.V.); enigmee@rambler.ru (N.D.)

**Keywords:** myocardial infarction, left ventricular ejection fraction, left ventricular remodeling, cardiac magnetic resonance imaging, extracellular volume

## Abstract

**Background/Objectives:** This study aimed to evaluate the prognostic role of the extracellular volume (ECV) assessed by cardiac magnetic resonance imaging (MRI) in relation to systolic dysfunction and unfavorable remodeling of the left ventricular (LV) at 24 weeks after myocardial infarction (MI) and revascularization. **Methods:** The study included 154 patients aged 56 ± 8 years who had been diagnosed with their first MI. Cardiac MRI was performed at 7–10 days and after 24 weeks, including assessment of indexed volumes, LV ejection fraction (LVEF), ECV, and patterns of ischemic and reperfusion injury. The study is registered in the international clinical trials registry with the number NCT04347434 (ClinicalTrials.gov). **Results:** Patients were divided into two groups after 24 weeks: group 1 (*n* = 24) with LVEF < 50% and group 2 (*n* = 130) with LVEF ≥ 50%. At 7–10 days, the scar mass in group 1 was 58.5 (39.5; 69.8) g vs. 17.1 (9.2; 29.7) g in group 2 (*p* < 0.001); microvascular obstruction was present in 23 cases (95.8%) vs. 55 (42.3%) (*p* < 0.001). After 24 weeks, inter-group differences increased (*p* < 0.05). Global ECV and remote myocardial ECV were significantly higher in patients with LVEF < 50% both at 7–10 days and after 24 weeks compared to those with LVEF ≥ 50% (*p* < 0.05). Infarct zone ECV did not differ between groups. Systolic dysfunction after 24 weeks was predicted by global ECV > 38.3% (*p* < 0.001) and remote myocardial ECV > 33.6% (*p* = 0.008). Predictors of an increase in end-diastolic volume index > 12% after 24 weeks in the subgroup of patients with initial systolic dysfunction were global ECV > 42.8% (*p* = 0.017) and remote myocardial ECV > 35.6% (*p* = 0.012). **Conclusions:** Global ECV and ECV of the remote myocardium, which exceed a certain level established by a cardiac MRI in the acute stage of MI, are among the predictors of LVEF < 50% and unfavorable LV remodeling in the medium term.

## 1. Introduction

Myocardial infarction (MI) remains the leading cause of heart failure (HF) and death worldwide. The process of left ventricular (LV) remodeling after MI is a key factor in the development of post-infarction HF. This process is characterized by the progressive dilation of the LV cavity and deterioration of its contractile function [1,2,3]. Despite the use of modern strategies for reperfusion and drug therapy after MI, a significant number of patients still develop HF due to adverse remodeling. This makes it essential to search for early and accurate markers of prognosis and to conduct in-depth studies of the mechanisms underlying this process in order to develop effective treatment strategies [4,5,6].

Traditionally, MI has focused on estimating the size of the necrosis zone and the contractile reserve [7]. It has been demonstrated that, along with LV ejection fraction (LVEF), a comprehensive assessment of the parameters of ischemic and reperfusion injury of the myocardium according to cardiac magnetic resonance imaging (MRI) data, such as the infarct zone, microvascular obstruction (MVO) and intramyocardial hemorrhage (IMH), makes it possible to better stratify the risk of cardiovascular complications after MI with ST-segment elevation and primary percutaneous coronary intervention [8]. At the same time, more and more data indicate the critical role of diffuse interstitial myocardial fibrosis, which precedes or develops after MI, as an independent substrate for LV myocardial dysfunction and electrical instability [9,10]. Cardiac MRI with contrast enhancement and quantification of extracellular volume (ECV) has become the “gold standard” for noninvasive assessment of both focal and diffuse fibrosis in vivo during the post-infarction period [11,12,13,14]. It has been established that in the acute stage of MI, elevated ECV acts as an integral marker of acute injury, summing up the contribution of edema, necrosis, and inflammation [15,16], and predicting the risk of MACE [17,18].

Despite this growing interest, the contribution of fibrosis in the infarcted zone and remote myocardium to chronic HF and long-term remodeling remains unclear. Additionally, specific ECV thresholds that have prognostic value after MI have not been fully elucidated.

The aim of the study was to evaluate the prognostic role of ECV in the infarction zone and the remote myocardium, determined by cardiac MRI, in relation to systolic dysfunction and unfavorable LV remodeling at week 24 after MI and revascularization.

## 2. Materials and Methods

A prospective cohort study was conducted, including 154 patients diagnosed with their first MI, confirmed according to clinical guidelines [19,20,21].

The present study is a secondary analysis of large-scale research, the registration number of which is in the international registry of clinical trials—NCT04347434 (ClinicalTrials.gov). The local ethics committee approved the study protocol and primary documentation on 27 September 2019.

Non-inclusion criteria: Contraindications to cardiac MRI, stenosis of the left main coronary artery > 30%, acute HF according to Killip class 3–4, chronic HF of functional class III-IV in the anamnesis, permanent atrial fibrillation, chronic kidney disease (glomerular filtration rate < 30 mL/min/1.73 m^2^), and other severe concomitant diseases. All patients signed an informed consent form.

Meeting the inclusion criteria and absence of non-inclusion criteria were confirmed in all patients. All 154 people underwent cardiac MRI at baseline and after 24 weeks.

The standard MRI protocol (GE SIGNA Voyager 1.5 Tl, GE HealthCare, Chicago, IL, USA) was performed on days 7–10 after MI and 24 weeks later, including an assessment of indexed volume parameters, global and local LV systolic function, local contractility index (LCI), and myocardial fibrosis using T1 mapping. Native and post-contrast T1 mapping was performed using the MOLLI method before and 15 min after administration of gadoteric acid (Clariscan™, GE Healthcare, Oslo, Norway) (0.15 mmol/kg) with an assessment of 16 segments. The ECV index was calculated using the formula: ECV = (1 − synthetic hematocrit) × ∆R1 of the myocardium/∆R1 of the blood. The synthetic hematocrit was determined by the T1 value of the blood pool, which made it possible to avoid delays between blood collection and scanning and minimize the effect of hemodilution on ECV accuracy [22]. ECV detection is a standard technique implemented in the certified software of an MRI machine and described in a number of publications that have demonstrated high inter- and intra-observational reproducibility [22,23].

ECV analysis identified regions of interest: 1—infarct zone (area of late gadolinium enhancement in the perfusion area of the infarct-related artery), 2—remote myocardium (visually intact myocardium in segments without late gadolinium enhancement), 3—global ECV (weighted average ECV value for the entire LV myocardium). The pattern of ischemic and reperfusion injury was determined by the following indicators: the mass of the scar and the peri-infarct zone (PIZ) heterogeneity, their ratio to the LV myocardial mass (LVMM), the ratio of the PIZ mass to the mass of the scar, the presence of microvascular obstruction (MVO) and intramyocardial hemorrhage (IMH).

The main endpoints after 24 weeks were systolic dysfunction (LVEF < 50%) and pathological LV dilation due to an increase in the end-diastolic volume index (EDVi) > 12% of the baseline [24].

Statistical analysis was performed in IBM SPSS Statistics v26.0 (IBM, Armonk, NY, USA). The normality of the data distribution was checked using the Shapiro–Wilk test. Quantitative data are presented as M ± SD with a normal distribution and Me (Q25; Q75) with an asymmetric distribution. For comparison, Student’s *t*-test and the Mann–Whitney U–test were used, respectively. Qualitative data is indicated as *n* (%); the comparison was performed using χ^2^. The relationships were evaluated by the Spearman correlation coefficient. Linear regression analysis with the indication of the regression coefficient β and 95% confidence interval (CI), logistic regression with the determination of the odds ratio (OR), and 95% CI were used for modeling. The prognostic value of ECV was assessed using ROC analysis. A significance level of *p* < 0.05 was used.

## 3. Results

The average age of the participants was 56 ± 8 years, with 138 men (89.6%) and 16 women (10.4%). ST-segment elevation MI was diagnosed in 136 people (88.3%), while non-ST-segment elevation MI was present in 18 individuals (11.7%). Primary percutaneous coronary intervention was performed in 106 (68.8%) patients, 48 (31.2%) persons underwent pharmacoinvasive reperfusion.

**Figure 1:**
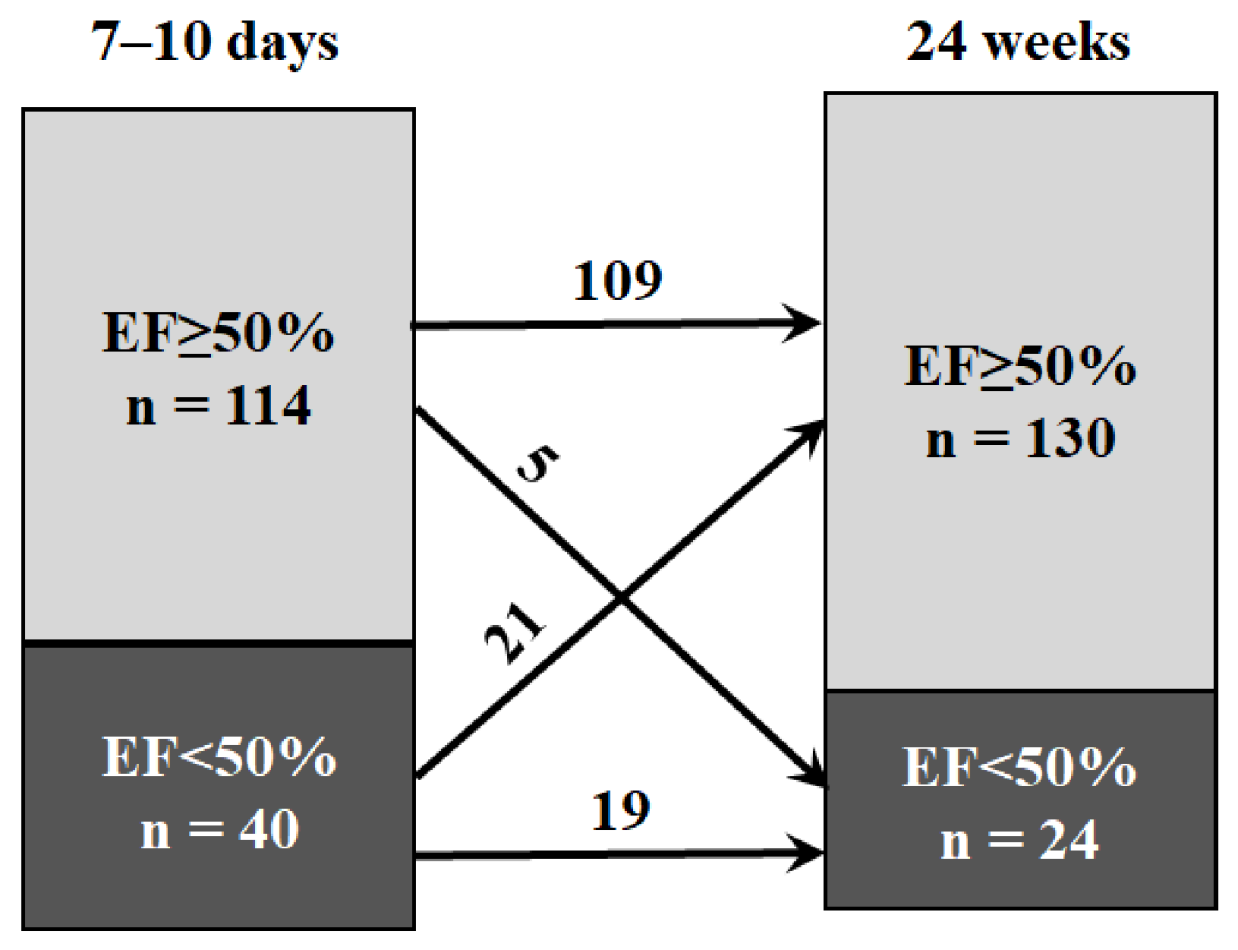
LVEF dynamics in the groups over 24 weeks.

Depending on the presence of systolic dysfunction, patients were divided into groups after 24 weeks: group 1 (*n* = 24; 15.6%) consisted of patients with LVEF < 50%, group 2 (*n* = 130; 84.4%) were patients with LVEF ≥ 50%. At the same time, on days 7–10, systolic dysfunction was observed in 40 people (26%). The intra-group dynamics of LVEF are shown in Figure 1.

The groups of patients with preserved and decreased systolic function 24 weeks after MI were comparable in terms of the main clinical and demographic parameters, which indicates the uniformity of the compared samples and the absence of influence of these factors on the identified differences (Table 1). In group 1, Q-MI was more common and the level of high-sensitivity troponin I was higher (*p* < 0.001), which indicates more severe myocardial damage at the onset of the disease.

The groups did not differ in terms of medications taken both during hospitalization (on days 7–10) and after 24 weeks (Table 2).

A comparative analysis of the LV volume, structural, and functional parameters, as well as the quantitative characteristics of ischemic and reperfusion injury over the 24-week follow-up period, is summarized in Table 3. As expected, on days 7–10, patients in group 1 demonstrated significantly lower LVEF (*p* < 0.001). The most prominent inter-group differences involved structural remodeling: in group 1, there was pronounced dilation of the LV cavity and a higher LVMM index.

Comparative analysis of the ischemic and reperfusion injury characteristics in the myocardium on days 7–10 also revealed that LVEF < 50% after 24 weeks is associated with more extensive post-infarction damage. In group 1, both the scar mass and its relation to LVMM were significantly higher. Although the mass of PIZ heterogeneity was reasonably higher in the systolic dysfunction group (*p* < 0.001), however, the ratio of the mass of PIZ heterogeneity to scar mass was higher in group 2 (*p* = 0.007), which probably indicates a greater potential for myocardial recovery in this group. Patients in group 2 were 2.3 and 3.9 times more likely to have MVO and IMH, respectively (*p* < 0.001), which confirms a more severe course of MI.

After 24 weeks in the LVEF group ≥ 50%, most of the cardiac MRI parameters improved. In particular, LVEF significantly increased, and the LVMM index and LCI decreased. Regression of most characteristics of ischemic injury was recorded, and the frequency of MVO and IMH decreased. At the same time, inter-group dynamics were more modest in patients with LVEF < 50%. The regression of only WMSI and the frequency of MVO was revealed. As a result, the differences between the groups increased after 24 weeks.

**Figure 2:**
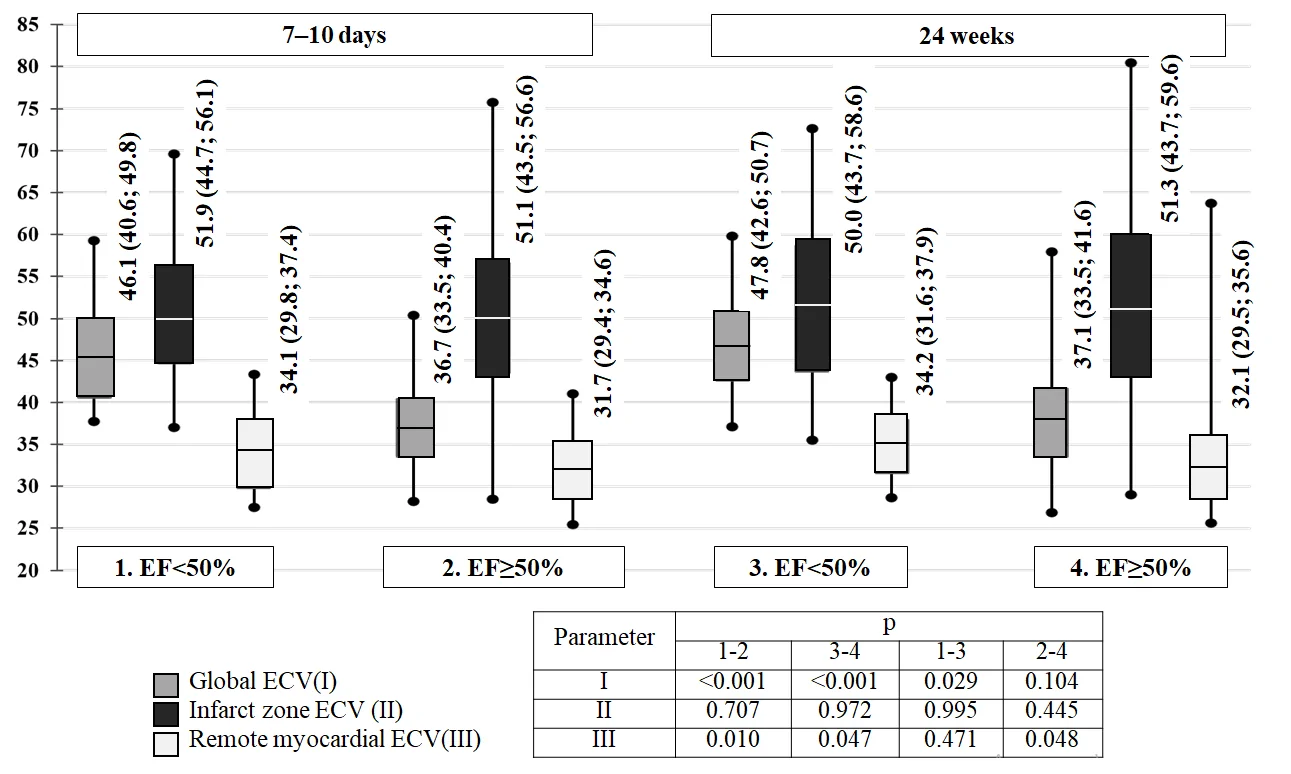
Comparison of ECV parameters in patients with preserved and reduced LVEF at 7–10 days and after 24 weeks after MI.

Global ECV and value of ECV of the remote myocardium showed significant differences in groups with reduced and preserved LV systolic function, both in the acute period of MI and after 24 weeks (Figure 2). Global ECV in patients of group 1 was higher both on days 7–10 and after 24 weeks (*p* < 0.001), with an additional increase from early to late. Thus, increased global ECV is associated with decreased EF and may be a marker of intercellular fluid accumulation in the early period and fibrosis in the late period, which has a negative effect on LV myocardial contractility.

Importantly, infarct zone ECV did not differ significantly between groups at either time point or in the inter-group analysis. Remote myocardial ECV was higher in group 1 at both early and late assessments, despite an increase in this parameter in group 2 at 24 weeks (*p* = 0.048). Thus, changes in the remote myocardium deserve attention as they may reflect diffuse interstitial fibrosis affecting LV function.

According to the results of the correlation analysis, the effect of global ECV on systolic heart function after 24 weeks of monitoring was manifested in a moderate negative association of the indicator with LVEF (r = –0.65; *p* < 0.001) as well as a moderate positive association with EDVi (r = 0.52; *p* < 0.001).

Linear regression analysis demonstrated a significant negative effect of global ECV measured at 7–10 days on LVEF after 24 weeks (β = –0.67 ± 0.08; [95% CI −0.82; −0.51]; *p* < 0.001). Logistic regression showed that this marker of myocardial fibrosis was also associated with subsequent LVEF < 50% after 24 weeks of supervision (OR 1.28 [95% CI 1.16; 1.42]; *p* < 0.001).

**Figure 3:**
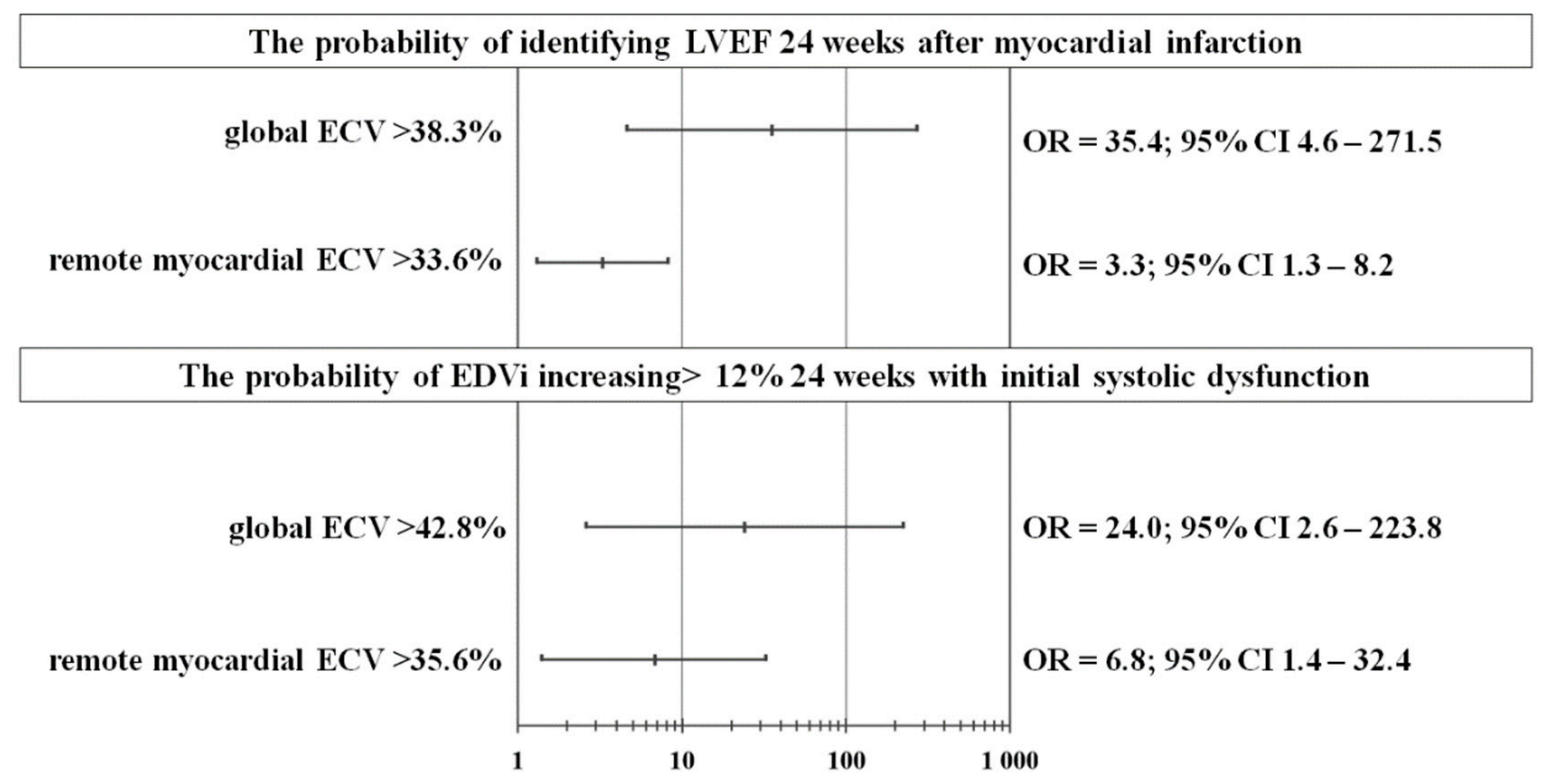
Predictive value of ECV parameters for identifying LVEF < 50% and LV dilation 24 weeks after MI. Note: CI—confidence interval; OR—odds ratio.

**Figure 4:**
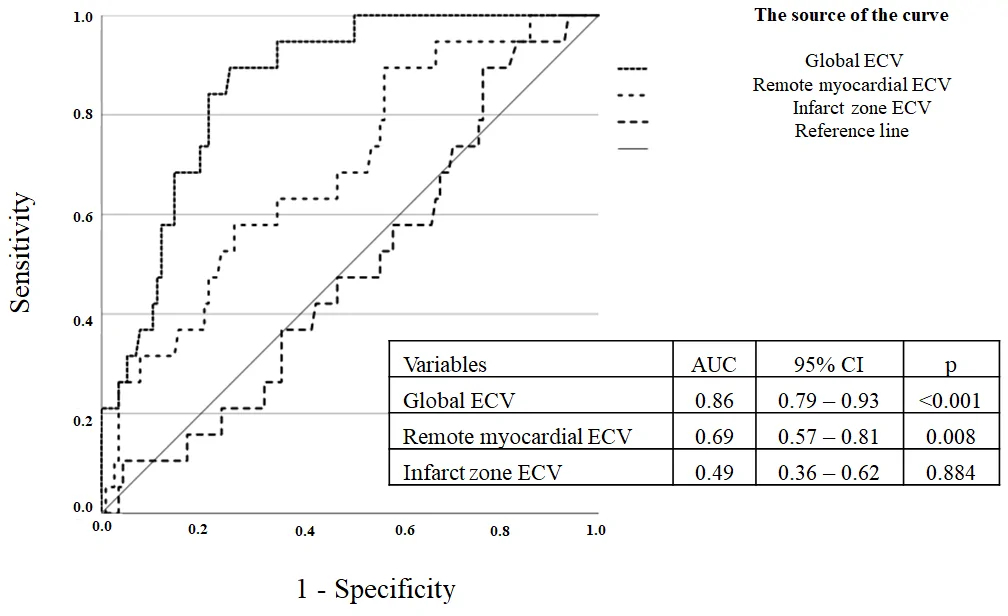
Predictive value of ECV parameters for identifying LVEF < 50% 24 weeks after MI. Note: AUC—area under the curve; CI—confidence interval; *p*—significance.

**Figure 5:**
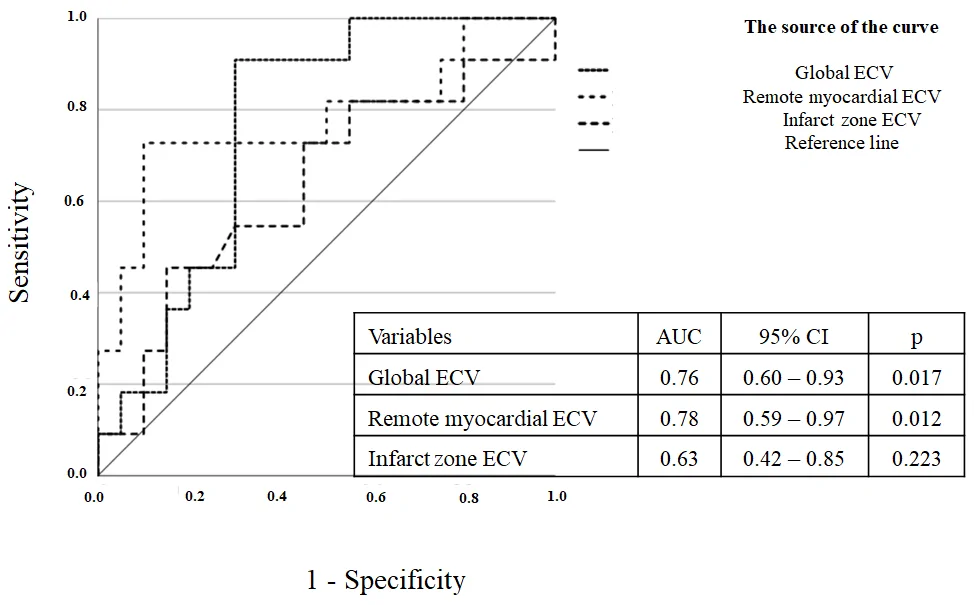
Predictive value of ECV parameters for identifying LV dilation 24 weeks after MI in the subgroup of patients with initial systolic dysfunction. Note: AUC—area under the curve; CI—confidence interval; *p*—significance.

ROC analysis confirmed the high prognostic value of early global ECV for LVEF < 50% after 24 weeks after MI, with a cut-off > 38.3% (sensitivity (Se) 95.7%, specificity (Sp) 62.5%) (Figure 3 and Figure 4). Exceeding this threshold was associated with a 35-fold increased likelihood of LVEF < 50% after 24 weeks (*p* = 0.001). Furthermore, in the subgroup of patients with initial systolic dysfunction (*n* = 40), global ECV predicted an increase in EDVi > 12% after 24 weeks (Figure 5), increasing the probability of this event 24-fold at a value > 42.8% (sensitivity 92.3%, specificity 66.7%) (Figure 4).

Linear regression analysis also showed a negative effect of the mean ECV value of the uninjured myocardium on the LVEF value after 24 weeks (β = −0.41 ± 0.16; [95% CI −0.72; −0.10]; *p* < 0.001), while baseline mean infarct zone ECV had no prognostic value (*p* = 0.226). Binary logistic regression confirmed a clear connection between mean remote myocardial ECV and LVEF < 50% after 24 weeks (OR 1.16 [95% CI 1.02 to 1.31]; *p* = 0.019), whereas baseline mean infarct zone ECV did not have such an effect (*p* = 0.717).

ROC analysis established that remote myocardial ECV measured at 7–10 days predicted detection of LVEF < 50% after 24 weeks after MI (Figure 3 and Figure 5), with no significant contribution from infarct zone ECV. A remote myocardial ECV threshold > 33.6% (Se 87.5%, Sp 43.7%) was associated with a 3.3-fold increased risk of LVEF < 50% after 24 weeks (*p* = 0.011). In the subgroup with initial systolic dysfunction, a predictor of EDVi increase > 12% after 24 weeks was remote myocardial ECV at 7–10 days (Figure 5); at a value > 35.6% (Se 61.5%, Sp 85.7%), the odds of developing dilation increased 6.8-fold (*p* = 0.016).

## 4. Discussion

EF remains a key measure of LV contractile function in patients with various cardiovascular diseases, including MI. Its ease of measurement, together with the proven connection between systolic dysfunction and a high rate of post-infarction damage, has led to its widespread clinical use for prognosis and treatment decisions [25,26]. The incidence in patients with moderately reduced and low LVEF after MI ranges from 25 to 38% [25,27]. In the present study, LVEF < 50% was diagnosed in 26% of patients at 7–10 days and in only 16% at 24 weeks. The difference is obviously due to the favorable course of the recovery period.

Previous studies have demonstrated a significant contribution of ischemic injury pattern characteristics to LVEF both in the acute stage and in the late post-infarction period [27,28,29]. Reperfusion injury also plays an important role in the development of systolic dysfunction [27,28,30,31]. It has been shown that patients with LVEF ≥ 50% after 24 weeks after ST-segment elevation MI have a reduction in scar mass, PIZ heterogeneity, MVO, and IMH, in contrast to those with LVEF < 50% [27]. In the presented study, patients with LVEF < 50% after 24 weeks were also characterized by unfavorable values of infarct zones in the early and late post-infarction periods: a greater scar mass and heterogeneous zone and a high frequency of different phenotypes of reperfusion injury, as well as the predominance of Q-MI on the electrocardiogram.

Myocardial fibrosis, resulting from increased myofibroblast activity and excessive extracellular matrix deposition, is a major component of myocardial remodeling and HF [32,33,34]. The differences in infarct zone characteristics between the comparison groups provide a basis for analyzing ECV parameters. In particular, a large scar mass and the presence of MVO and IMH (including those characterizing chronic iron deposition in the long-term period of MI) predispose to higher ECV values in the infarction zone, and a large mass of myocardial hypertrophy can affect global ECV and ECV of the remote myocardium, forming a substrate for extensive fibrosis. Thus, the remote myocardium cannot be considered intact since edema, inflammation, microvascular injury, and apoptosis of cardiomyocytes may persist in it, which are not detected by visual assessment using the local hyperperfusion method [35,36].

Two main types of myocardial fibrosis are recognized: replacement (reparative) and interstitial (reactive and infiltrative) [32,37]. Reparative fibrosis represents irreversible collagen deposition after cardiomyocyte necrosis and plays a crucial role in preventing LV wall rupture. In contrast, reactive fibrosis in the remote uninjured myocardium occurs due to chronic hemodynamic overload, ultimately leading to progressive global LV contractile function and HF development [37,38,39,40]. In this study, as early as 7–10 days, patients who later had LVEF < 50% after 24 weeks already showed higher global ECV and remote myocardial ECV, confirming that the fibrotic process is not confined to the necrotic zone but involves the entire myocardium and that the state of remote segments plays a dominant role in determining global contractility. Meanwhile, the value of ECV in the infarct zone did not differ between groups with different LVEF, indicating its limited prognostic value for global systolic function compared with the remote myocardium. A comparative assessment of the contribution of ECV parameters during regression analysis confirmed its effect in the remote myocardium, but not in the infarct zone ECV, on systolic function and LV dilation at 24 weeks after MI. Thus, the key finding of the study is the confirmation of the critical role of diffuse interstitial fibrosis, assessed by remote myocardial ECV, as the leading predictor of LV systolic dysfunction and post-infarction LV remodeling in the medium term, compared to replacement fibrosis in the necrotic area [41].

The lack of a significant connection between ECV infarct zone and the development of LV remodeling and dysfunction has a clear pathophysiological explanation. The primary response to MI involves a cascade of events including release of matrix metalloproteinases and proteases, which trigger an inflammatory reaction in the injured area and degrade the extracellular matrix after the proliferative phase. Subsequently, collagen types I and III are deposited in the necrotic zone, forming a fibrous scar [42,43,44]. Since cardiomyocytes are completely lost in the necrosis zone, a further increase in the density of the collagen matrix does not have an additional negative effect on global systolic function, which is primarily limited by scar size rather than its qualitative characteristics. In contrast, increased ECV in the remote myocardium, reflecting collagen accumulation among viable cardiomyocytes, directly impairs their contractile ability and increases myocardial stiffness, explaining its role as a predictor of systolic dysfunction and LV dilation [38]. Indeed, pathological remodeling involves fibroblast-mediated expansion of the extracellular matrix, compensatory cardiomyocyte hypertrophy in response to increased LV wall stress, increased wall stiffness due to excessive collagen, and tonic contraction of fibrous tissue, leading to diastolic dysfunction and HF [4,10,38,44].

The presented results are of great clinical significance, as the identification of increased values of global ECV and ECV in the remote myocardium will enable risk re-stratification followed by active dynamic monitoring and optimization of drug treatment. In particular, the obtained threshold values of global ECV for predicting systolic dysfunction and unfavorable remodeling, as well as remote myocardial ECV for predicting systolic dysfunction, prioritize sensitivity over specificity, reflecting their aim to minimize false-negative results. Consequently, these criteria can be integrated into early risk stratification algorithms for patients after MI. In contrast, the remote myocardial ECV threshold for predicting LV dilation showed moderate sensitivity combined with reasonably high specificity, indicating that this parameter can confirm the risk of unfavorable remodeling with acceptable diagnostic accuracy. Given the potential reversibility of diffuse interstitial fibrosis in its early stages, identifying such patients opens a therapeutic window for intensifying pathogenetic pharmacotherapy and, ultimately, for using antifibrotic drugs to prevent progressive LV remodeling and systolic dysfunction.

## 5. The Current State of the Problem

We have compared our results with previous studies on the remote myocardium after MI with ST-segment elevation. Our findings confirm the previously known patterns and provide new data. First and foremost, our results align with the work of Bulluck et al. and Carberry et al. [45,46], who have shown that native T1 and ECV in areas remote from the infarction already increase during the acute phase and persist in the subacute period. These changes are indicative of diffuse interstitial edema and subsequent reactive fibrosis, which we have also observed. In the present study, we found that the ECV of the remote myocardium was significantly higher in patients with LVEF < 50% after 24 weeks.

The presented data support the findings of Garg et al., which state that the volume of scar tissue correlates positively with the severity of changes in the remote myocardium [47]. The larger the necrosis, the stronger the neurohormonal stimulus for remodeling the entire left ventricle. According to Black et al., this relationship has been described as linear [48]. However, our findings suggest that with a large scar volume, the ECV gain of the remote myocardium becomes disproportionately high, potentially indicating a threshold effect on fibrogenesis activation.

We did not find a significant correlation between the ECV of the remote myocardium and local wall thinning in these segments. This is an important difference compared to a number of previous studies (Messroghli et al., 2017 [49]). It seems that a decrease in myocardial thickness is a late and more obvious sign of myocardial remodeling, while increased ECV is an early indicator of interstitial abnormalities even before macroscopic changes in the wall occur.

Finally, with regard to LV remodeling, our results are closest to those of Carberry et al. [46]. In this study, ECV in the remote myocardium predicted an increase in EDV after 6 months. In turn, we have shown that ECV of the remote myocardium is independently associated with systolic dysfunction after 6 months. This predictive ability persists even after adjusting for the initial LV and EF volumes.

Thus, the presented study confirms that the remote myocardium actively affects the course of the post-infarction period. The quantitative assessment of ECV contains important independent prognostic information regardless of the size of infarction and reperfusion injury. Therefore, the ECV indicator can potentially be considered as an indicator of monitoring the effectiveness of antifibrotic therapy.

### Study Limitations

This single-center study included only patients diagnosed with their first MI after reperfusion, which may limit extrapolation to other patient populations, such as those with recurrent MI or older age groups. It is also worth noting the small number of adverse outcomes and the wide confidence intervals for the calculated odds ratios. This may be related to the small sample size and suggests the need for additional prospective studies to validate the proposed ECV thresholds externally. The significant male predominance in the study is typical of most original works on MI and reflects the actual ratio of men to women with the disease under the age of 70.

## 6. Conclusions

Patients with LVEF < 50% at 24 weeks after MI had unfavorable patterns of ischemic and reperfusion injury and predominance of global ECV and ECV in the remote myocardium throughout the observation period. Global ECV measured in the acute stage of MI is one of the predictors of LVEF < 50% and unfavorable LV remodeling in the medium term. The prognostic value of ECV in the remote myocardium substantially exceeds that of infarct zone ECV, indicating the key pathogenetic role of diffuse interstitial fibrosis in remote segments as one of the main drivers of systolic dysfunction and progressive LV dilation in the long-term post-infarction period. Conversely, the degree of fibrosis within the necrotic zone itself has no independent prognostic value for global cardiac function.

## Figures and Tables

**Table 1:** Clinical and demographic characteristics of groups with preserved ejection fraction and with systolic dysfunction 24 weeks after MI.

Parameter	LVEF < 50%, *n* = 24	LVEF ≥ 50%, *n* = 130	*p*
Age, years	56.3 ± 8.2	55.6 ± 7.7	0.675
Male, *n* (%)	22 (91.7)	116 (89.2)	0.719
Female, *n* (%)	2 (8.3)	14 (10.8)	0.719
Body mass index, kg/m^2^	27.9 ± 3.8	28.0 ± 3.9	0.954
Body mass index ≥ 30 kg/m^2^, *n* (%)	8 (33.3)	31(23.8)	0.615
Prior angina, *n* (%)	7 (29.2)	26 (20.0)	0.315
Hypertension, *n* (%)	21 (87.5)	110 (84.6)	0.716
Type 2 diabetes mellitus, *n* (%)	1 (4.2)	11 (8.5)	0.471
Smoking, *n* (%)	15 (62.5)	99 (76.2)	0.161
ST-segment elevation MI, *n* (%)	23 (95.8)	113 (86.9)	0.212
Q-wave MI, *n* (%)	21 (87.5)	67 (51.5)	0.001
Pharmacoinvasive reperfusion, *n* (%)	10 (41.7)	38 (29.2)	0.227
Primary percutaneous coronary intervention, *n* (%)	14 (58.3)	92 (70.8)	0.381
The time of “pain—thrombolysis”, min	115 (83; 350)	90 (41;158)	0.408
The time of “pain—stent”, min	310 (203; 682)	275 (170; 496)	0.655
High-sensitivity troponin I, ng/mL	78.8 (29.4; 159.3)	24.8 (7.5; 50.0)	<0.001

Note: LVEF—left ventricular ejection fraction; MI—myocardial infarction; *n*—number of patients; *p*—significance.

**Table 2:** Medication therapy for 24 weeks in comparison groups.

Group of Medications	Visit	LVEF < 50%, *n* = 24	LVEF ≥ 50%, *n* = 130	*p*1–2
		(1)	(2)	
Dual Antiplatelet Therapy, *n* (%)	7–10 days	24 (100)	130 (100)	1.000
	24 weeks	24 (100)	130 (100)	1.000
Statins, *n* (%)	7–10 days	24 (100)	130 (100)	1.000
	24 weeks	24 (100)	130 (100)	1.000
Ezetimibe, *n* (%)	7–10 days	0 (0)	2 (1.5)	0.541
	24 weeks	19 (79.2)	89 (68.5)	0.292
Beta-blockers, *n* (%)	7–10 days	23 (95.8)	117 (90)	0.361
	24 weeks	18 (75)	98 (75.4)	0.968
Renin–angiotensin–aldosterone system blockers, *n* (%)	7–10 days	22 (91.7)	116 (89.2)	0.719
	24 weeks	19 (79.2)	103 (79.2)	0.994
Calcium channel blockers, *n* (%)	7–10 days	2 (8.3)	13 (10)	0.800
	24 weeks	2 (8.3)	14 (10.8)	0.719
Diuretics, *n* (%)	7–10 days	15 (62.5)	54 (41.5)	0.058
	24 weeks	13 (54.2)	48 (36.9)	0.113
Sodium-glucose cotransporter 2 inhibitors, *n* (%)	7–10 days	2 (8.3)	10 (7.7)	0.914
	24 weeks	11 (45.8)	46 (35.4)	0.330

**Table 3:** Dynamics of left ventricular parameters and characteristics of ischemic and reperfusion injury over 24 weeks in comparison groups.

Parameter	Visit	LVEF < 50%, *n* = 24	LVEF ≥ 50%, *n* = 130	*p*1–2
		(1)	(2)	
LVEF, %	7–10 days	43.3 (40.3; 48.9)	55.0 (51.5; 58.5)	<0.001
	24 weeks	44.1 (40.7; 47.3)	57.6 (54.1; 61.3) *	<0.001
EDVi, ml/m^2^	7–10 days	93.5 (78.3; 120.1)	80.6 (67.2; 89.8)	<0.001
	24 weeks	109.3 (89.0; 123.4) *	81.7 (73.2; 90.6) *	<0.001
EDVi increasing > 12%, *n* (%)	24 weeks	10 (41.7)	39 (30)	0.260
ESVi, ml/m^2^	7–10 days	52.8 (42.7; 71.8)	34.9 (28.7; 41.2)	<0.001
	24 weeks	61.3 (45.1; 71.6)	34.9 (29.0; 39.5)	<0.001
ESVi decreasing > 12%, *n* (%)	24 weeks	4 (16.7)	37 (28.5)	0.230
LVMM index, g/m^2^	7–10 days	64.2 (58.5; 72.4)	57.5 (53.0; 63.7)	0.002
	24 weeks	65.0 (62.0; 70.9)	56.4 (52.1; 61.7) *	<0.001
LCI	7–10 days	2.3 (2.1; 2.5)	1.4 (1.2; 1.7)	<0.001
	24 weeks	2.1 (1.9; 2.3) *	1.2 (1.0; 1.4) *	<0.001
Scar mass, g	7–10 days	58.5 (39.5; 69.8)	17.1 (9.2; 29.7)	<0.001
	24 weeks	45.6 (33.5; 63.3)	13.2 (7.6; 22.5) *	<0.001
Scar mass/LVMM, %	7–10 days	42.2 (30.5; 53.1)	15.1 (7.4; 23.0)	<0.001
	24 weeks	38.1 (25.4; 49.4)	12.1 (6.2; 20.2) *	<0.001
PIZ mass, g	7–10 days	18.6 (13.0; 27.2)	11.2 (6.6; 15.9)	<0.001
	24 weeks	16.9 (9.3; 21.5)	8.3 (5.1; 14.3) *	<0.001
PIZ mass/scar mass, %	7–10 days	38.7 (24.9; 60.1)	67.2 (44.0; 100.3)	0.007
	24 weeks	41.7 (22.1; 49.5)	62.4 (43.1; 100.4)	<0.001
MVO, *n* (%)	7–10 days	23 (95.8)	55 (42.3)	<0.001
	24 weeks	16 (66.7%) *	23 (17.7%) *	<0.001
IMH, *n* (%)	7–10 days	13 (54.2)	18 (13.8)	<0.001
	24 weeks **	12 (50.0%)	5 (3.8%) *	<0.001

Note: * *p* < 0.05—intra-group dynamics, **—the IMH index after 24 weeks reflects chronic iron deposition, EDVi—end-diastolic volume index; ESVi—end-systolic volume index; IMH—intramyocardial hemorrhage; LVEF—left ventricular ejection fraction; LVMM—left ventricular myocardial mass, MVO—microvascular obstruction, PIZ—peri-infarct zone heterogeneity, *p*—significance.

## Data Availability

The original contributions presented in this study are included in the article. Further inquiries can be directed to the corresponding author.
